# Phlebotomine sand flies (Diptera, Psychodidae) from Pha Tong cave, Northern Thailand with a description of two new species and taxonomical thoughts about *Phlebotomus stantoni*

**DOI:** 10.1371/journal.pntd.0011565

**Published:** 2023-09-20

**Authors:** Marie-Charlotte Renaux Torres, Coline Pellot, Puckavadee Somwang, Pathamet Khositharattanakool, Khamsing Vongphayloth, Fano José Randrianambinintsoa, Bruno Mathieu, Padet Siriyasatien, Frédérick Gay, Jérôme Depaquit

**Affiliations:** 1 Faculté de Pharmacie, Université de Reims Champagne-Ardenne, SFR Cap Santé, EA7510 ESCAPE-USC ANSES PETARD, Reims, France; 2 Pharmacie centrale, Centre Hospitalo-Universitaire, Reims, France; 3 School of Medicine, Mae Fah Luang University, Chiang Rai, Thailand; 4 Biomedical Technology Research Group for Vulnerable Populations, Mae Fah Luang University, Chiang Rai, Thailand; 5 Pasteur Institute of Laos, Vientiane, Lao PDR; 6 Institut de Parasitologie et de Pathologie Tropicale de Strasbourg, Université de Strasbourg, Strasbourg, France; 7 Center of Excellence in Vector Biology and Vector-Borne Disease, Department of Parasitology, Faculty of Medicine, Chulalongkorn University, Bangkok, Thailand; 8 Sorbonne Université, INSERM, Institut Pierre Louis d’Epidémiologie et de Santé Publique, Hôpital Pitié Salpêtrière, APHP, F75013, Paris, France; 9 Centre Hospitalo-Universitaire, pôle de Biologie territorial, Laboratoire de Parasitologie- Mycologie, Reims, France; University of Calgary, CANADA

## Abstract

**Background:**

In South-East Asia, Thailand is the country with the highest number of human autochthonous cases of leishmaniases mostly due to *Leishmania martiniquensis*. Their transmission remains unresolved to date even though sand flies are known vectors of leishmaniases. As such, we focused a study on the sand fly fauna of a cave in Thailand to explore the biodiversity of potential *Leishmania* vectors.

**Main results:**

We carried out an inventory in Pha Tong cave. We caught and identified 570 Phlebotomine sand flies (452 females and 118 males) and identified 14 species belonging to the genera *Phlebotomus*, *Idiophlebotomus*, *Chinius*, *Sergentomyia* and *Grassomyia*. Among these 14 species, two could not be related to known sand fly species. Herein, we propose the description of two new sand fly species, previously unknown to science.

The first new species, *Phlebotomus shadenae* n. sp. is a sand fly of the subgenus *Anaphlebotomus*. It is morphologically close to *Ph*. *stantoni*, a species widely distributed throughout Southeast Asia. However, it differs by the length of the genital filaments in males or by the length of the ducts of the spermathecae in females as well as the high divergence of cytochrome b sequences. Additionally, we revised the systematics of the subgenus *Anaphlebotomus* and reinstated, by examination of its holotype, the validity of *Ph*. *maynei*, an Indian wrongly considered as a synonym of *Ph*. *stantoni* in the past.

The second new species, *Sergentomyia maiae* n. sp., differs from a species in the same group, *Se*. *barraudi*, by an original cibarial double row of vertical teeth as well as by molecular data.

**Conclusions:**

We propose the description of two new sand fly species for Science with morphological and molecular evidence. *Ph*. *shadenae* n. sp. was also found to be distributed in the south of Thailand and in Laos. Future studies need to determine whether these two species can play a role as vectors of *Leishmania* parasites, Trypanosomatids or Phlebovirus. Most of the species caught in the present study are strictly cavernicolous except *Grassomyia* sp. and a few *Sergentomyia*.

## Author summary

Phlebotomine sand flies are tiny blood sucking insects. These are vectors of *Leishmania*, flagellate unicellular parasites, mostly found in Mediterranean and tropical areas. In Thailand, *Leishmania martiniquensis* and *L*. *orientalis* are responsible for local diseases but the parasite transmission cycle remains unknown. For a long time, South-Eastern Asia was considered to be free of leishmaniasis and as such, Phlebotomine sand flies have been understudied in this area.

We carried out an inventory in a cave located in the Chiang Rai district, northern Thailand. We caught two new species we consider to be new for Science based on morphological (head and genital apparatus) and molecular characters (cytochrome b mitochondrial DNA sequences).

The new species were named *Phlebotomus shadenae* n. sp. and *Sergentomyia maiae* n. sp. We also recorded the first species in Laos whereas the second has just been recorded in the studied cave.

## 1 Introduction

Phlebotomine sand flies are small, hairy, blood-sucking insects belonging to the order Diptera and to the family Psychodidae. Sand flies are known as important medical vectors of leishmaniasis around the world. Leishmaniasis is a spectrum of neglected diseases, mostly distributed in Mediterranean and tropical areas, caused by the flagellate protozoa belonging to the genus *Leishmania*.

In Thailand, *Leishmania martiniquensis* and *L*. *orientalis* have been identified as the causative agents of autochthonous human visceral and cutaneous leishmaniasis. The disease is endemic mostly in southern Thailand but some cases have also been recorded in the northern part of the country. Though the first indigenous case of leishmaniasis was reported in Thailand 25 years ago [1], data on the sand fly fauna remains limited and their role in the transmission of *Leishmania* has not been proven yet. In Thailand, reports have identified four genera of sand flies to date: *Phlebotomus*, *Sergentomyia*, *Idiophlebotomus*, and *Chinius*.

We carried out an inventory in the Pha Tong cave, located in the Chiang Rai district, Northern Thailand. Several studies carried out in caves revealed an interesting sand fly biodiversity and new species as well as a high density of sand flies [2–7]. We identified several species belonging to the four genera previously listed and also to the genus *Grassomyia*. However, several specimens from this cave could not be identified according to the existing described species. We performed a morphological and molecular study, and based on their new characters, we herein propose the description of two new sand fly species for Science.

## 2 Methods

### 2.1. Sampling

During an entomological survey of cavernicolous sand flies in Asia, we carried out a sampling of Phlebotomine sand flies in the Pha Thong cave (20°5′28.89"N, 99°54′13.09"E), Chiang Rai province, Northern Thailand on October 10^th^ and 11^th^, 2019. These caves are listed as sights of interest and are visited by Thai and international tourists. Sand flies were collected over two consecutive nights using CDC miniature light traps (John W. Hock Company, Gainesville, FL, USA). Four traps were installed from 5 p.m. to 8 a.m. within the cave, at a distance less than 100 m from the cave entrance, in the dark area (completely removed from sunlight) with a constant average temperature of 25°C and an average relative humidity of 80%. Sand flies were caught alive, frozen and stored in 96% ethanol in the hours following their capture.

### 2.2. Morphological analysis

The head, thorax and genitalia of the sand flies were cut off and placed in a drop of ethanol. Soft tissues were lysed in a bath of 10% potassium hydroxide (KOH), then bleached in a Marc-André solution. After dehydration in successive alcoholic baths, these parts of the insects were then mounted between microscope slide and cover slide in Euparal for species identification. The first abdominal tergites of each specimen were dried before any processing and stored in a vial at -20°C for later molecular analysis. The specimens were observed with an Olympus BX50 microscope coupled with a DP 26 Olympus camera allowing photographs to be taken. Measurements and counting of several characters used in systematics [8] have been realized using Stream Essentials software (Olympus, Japan). The sketches were made using the camera lucida installed on the microscope. The drawings were then finalized using Indian ink.

### 2.3. Molecular analyses

Genomic DNA was extracted from the first abdominal tergites of individual sand flies using the QIAmp DNA Mini Kit (Qiagen, Germany) following the manufacturer’s instructions. The first step consisted in crushing the sand flies with a piston pellet (Treff, Switzerland). In the end, the elution volume was 100 μL. All polymerase chain reaction (PCR) amplifications were performed in a 50 μL volume of 5 μL extracted DNA and 50 pmol of each of the primers. The final concentrations of the PCR mix contained 10 mM Tris HCl (pH 8.3), 1.5 mM MgCl2, 50 mM KCl, 0.01% Triton X 100, 200 μM dNTP each base, and 1.25 units of 5 prime *Taq* polymerase (Eppendorf, Germany). The amplification of a fragment of the cytochrome b gene (cyt b) was undertaken using the primers C3B-PDR (5’- CAYATTCAACCWGAATGATA-3’) and N1N-PDR (5’-GGTAYWTTGCCTCGAWTTCGWTATGA-3’) according to the conditions previously published [9]. Amplicons were sequenced in both directions using the same primers.

### 2.4. Phylogenetic analyses

Molecular analyses were based on aligned sequences. The maximum likelihood (ML) tree was constructed by MEGA XI [10] using the HKY85 substitution model [11].

## 3. Results

### 3.1. Inventory

A total of 570 Phlebotomine sand flies were caught during sampling of this study. According to morphological identification, the collected sand flies belong to five genera (*Chinius*, *Idiophlebotomus*, *Phlebotomus*, *Sergentomyia* and *Grassomyia*) and 14 species (Table 1).

**Table 1:** Morphological identification of the specimens caught in the present study.

Genus	Species	Females	Males	Total
*Phlebotomus*	*barguesae*	4	1	5
	*mascomai*	4		4
	*stantoni*	102	41	143
	*shadenae* n. sp.	1	3	4
*Sergentomyia*	*anodontis*	178		178
	*hivernus*	2		2
	*cf iyengari* Raynal-like	1		1
	*khawi*	29		29
	*maiae n*. *sp*.	12		12
	*siamensis*	105		105
	*sylvatica*	1		1
	*spp*.	3	70	73
*Grassomyia*	*sp*.	4	3	7
*Chinius*	*barbazani*	3		3
*Idiophlebotomus*	*longiforceps*	3		3
Total		452	118	570

Two of these 14 species could not be identified to existing sand fly species and we consider them to be new Phlebotomine species for Science.

Given that *Sergentomyia* males are closely related, in order to avoid any misidentification between species, all the *Sergentomyia* males were inventoried as *Sergentomyia* spp.

### 3.2. Molecular analysis

**Fig 1:**
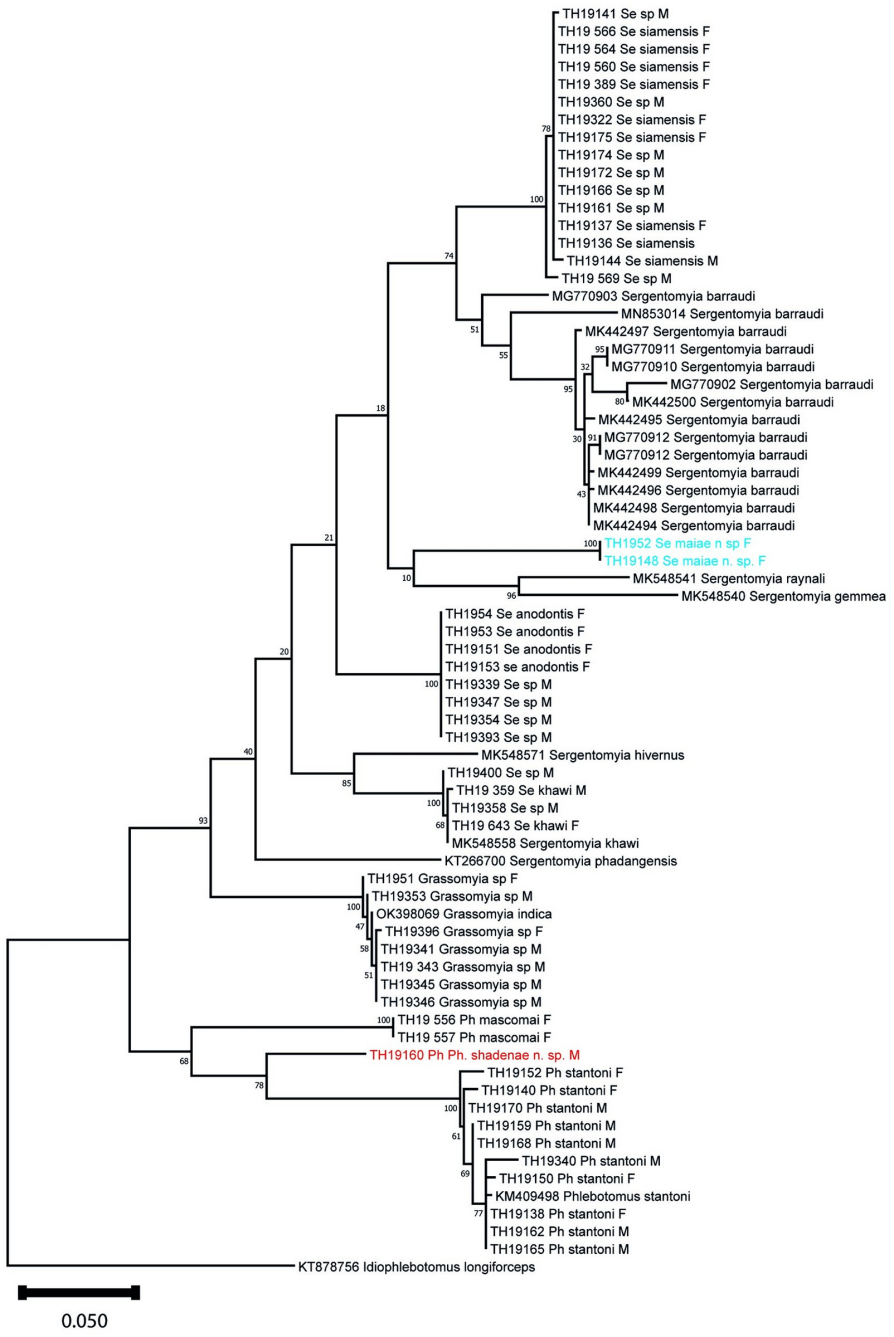
Maximum likelihood tree. The tree is inferred from an alignment of 488 bp of cyt b sequences by using a Hasegawa-Kishino-Yano 85 model. The tree was rooted on *Idiophlebotomus longiforceps*.

The 520 bp database includes 236 variable sites and 196 informative for parsimony. A maximum likelihood tree based on this database is shown in Fig 1. The estimation of evolutionary divergence over sequence pairs between and within species is provided in Table 2. Sequences obtained in the present study are available in Genbank (accession numbers OQ784691 to OQ784740).

**Table 2:** Number of base differences per site from averaging over all sequence pairs between species (thin style) and within species (bold style) calculated using a p-distance model on the cyt b dataset.

	*Grassomyia*	*Se*. *maiae n*. *sp*.	*Se*. *anodontis*	*Se*. *siamensis*	*Ph*. *stantoni*	*Ph*. *shadenae n*. *sp*.	*Se*. *khawi*	*Ph*. *mascomai*	*Se*. *hivernus*	*Se*. *raynali*	*Se*. *gemmea*	*Se*. *phadangensis*	*Se*. *barraudi*	*Id*. *longiforceps*
*Grassomyia sp*	**0.003**													
*Se*. *maiae n*. *sp*.	0.159	**0**												
*Se*. *anodontis*	0.124	0.122	**0**											
*Se*. *siamensis*	0.141	0.120	0.095	**0.002**										
*Ph*. *stantoni*	0.196	0.190	0.174	0.195	**0.012**									
*Ph*. *shadenae n*. *sp*.	0.155	0.166	0.145	0.174	0.123	**n/a**								
*Se*. *khawi*	0.126	0.128	0.099	0.126	0.191	0.167	**0.002**							
*Ph*. *mascomai*	0.173	0.188	0.157	0.182	0.161	0.132	0.175	**0**						
*Se*. *hivernus*	0.134	0.148	0.117	0.145	0.209	0.184	0.086	0.189	**n/a**					
*Se*. *raynali*	0.125	0.142	0.121	0.140	0.218	0.191	0.122	0.189	0.125	**n/a**				
*Se*. *gemmea*	0.148	0.162	0.137	0.156	0.236	0.210	0.123	0.225	0.148	0.107	**n/a**			
*Se*. *phadangensis*	0.143	0.157	0.130	0.130	0.188	0.165	0.128	0.164	0.137	0.152	0.182	**n/a**		
*Se*. *barraudi*	0.147	0.139	0.121	0.088	0.214	0.188	0.133	0.180	0.122	0.119	0.145	0.126	**0.03**	
*Id*. *longiforceps*	0.223	0.249	0.209	0.227	0.231	0.209	0.222	0.208	0.247	0.222	0.243	0.225	0.235	**n/a**

### 3.3. Description of new taxa

The consensual terminology was used in this description [12].

#### 3.3.1. Description of the female of *Ph*. *shadenae* Depaquit, Pellot and Siriyasatien *n*. *sp*. (Fig 2)

**Fig 2:**
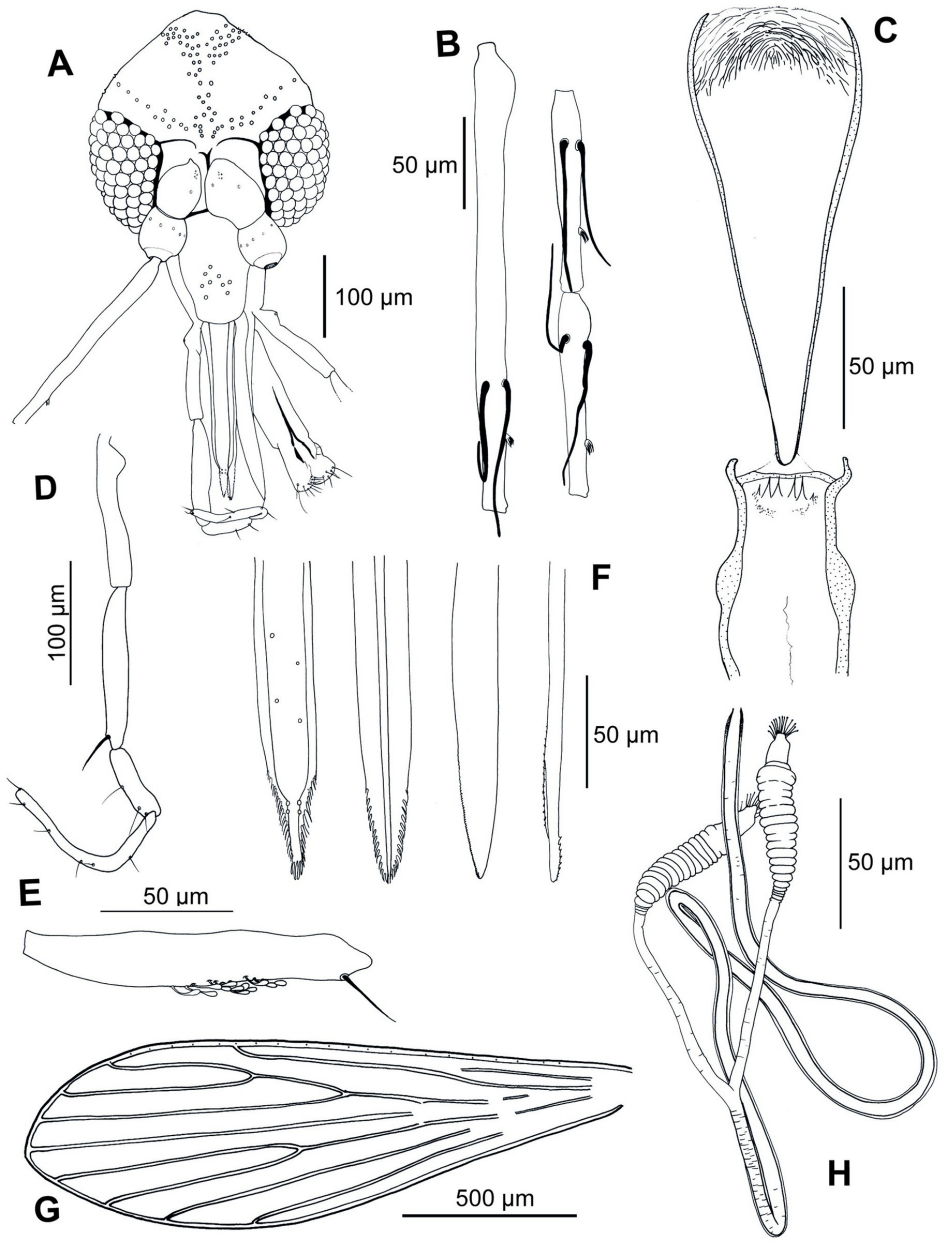
*Phlebotomus shadenae* n. sp. Holotype female. A: head; B: flagellomeres 1, 2 and 3 (= AIII, AIV and AV); C: pharynx and cibarium; D: palp; E: third segment of the palp (P3); F: mouth parts (labrum, hypopharynx, mandible and maxilla, from left to right); G: wing; H: spermathecae.

Genus *Phlebotomus* Rondani & Berté, in Rondani 1840.

Subgenus *Anaphlebotomus*, Theodor 1948.

Description of female holotype (specimen TH19-255). Comparative measurements of *Ph*. *shadenae* n. sp. with those of *Ph*. *stantoni* and *Ph*. *hoepplii* are indicated in Table 3.

**Table 3:** Asian *Anaphlebotomus* female measurements (in μm). Those of *Ph*. *stantoni* and *Ph*. *shadenae* n. sp. were done during the present study. Those of *Ph*. *hoepplii* were calculated from the original description of Tang & Maa (1945). *: estimated after the drawing of plate II by Tang & Maa (1945).

		*Ph*. *stantoni* (n = 12)				*Ph*. *shadenae n*. *sp*. holotype	*Ph*. *hoepplii* (n = 5)			
		mean	minimum	maximum	standard deviation		mean	minimum	maximum	standard deviation
Head	length	370.42	337.28	396.05	18.15	375.24				
	width	308.89	289.52	332.64	13.37	278.56				
Clypeus	length	120.83	90.08	148.56	17.56	137.47				
Flagellomeres	f1	257.96	236.6	283.37	12.71	256.28	251.8	236	271	15.63
	f2	92.11	83.01	100.44	6.46	105.03	105.4	98	114	5.68
	f3	96.26	84.6	105.74	7.37	111.34	105	96	114	6.36
Labrum	length	212.4	192.05	237.28	12.7	187.68				
Palpi	p1	33.25	25.11	41.82	4.95	27.75	31	27	40	5.87
	p2	87.19	75.17	97.96	6.84	81.06	90	72	99	10.56
	p3	130.66	117.73	149.1	9.3	125.55	130.2	117	144	10.08
	p4	55.13	40.84	64.15	7.43	58.39	56.2	50	63	4.82
	p5	159.04	113.71	182.92	23.79	154.01	109.6	72	188	46.08
Cibarium	teeth	3.75	2	5	0.97	4				
Wing	length	1684.02	1543.01	1821.25	88.35	1677.57	1862	1750	2066	131.68
	width	577.49	517.67	620.73	30.79	543.66	551.2	481	612	49.86
Spermathecae	common duct	218.75	178	261	28.32	508				
	individual ducts	51.75	43	63	7.81	69	20*			

***Etymology***: the epithet *shadenae* refers to our colleague Shaden Kamhawi.

Type locality: Pha Thong cave (20° 5′28.89"N, 99°54′13.09"E), Chiang Rai province, northern Thailand.

**Type specimens**: female holotype (voucher TH19-255) and one male paratype (voucher TH19-677) deposited at the Laboratory of Entomology of the Muséum National d’Histoire

Naturelle de Paris, France under the identification numbers MNHN-ED-ED11207 and MNHN-ED-ED11208.

One male paratype (voucher TH19-160) deposited at the Natural History Museum of the National Science Museum, Thailand, under the identification number THNHM-I-00028085.

**Note**: The authors of the new taxa are different from the authors of this paper: Article 50.1 and Recommendation 50A of the International Code of Zoological Nomenclature [13].

**3.3.1.1**. **Head**

Occiput with two narrow lines of well individualized setae. Clypeus: 135 μm long and 64 μm wide exhibiting nine setae.

Eyes: 158 μm long, 78 μm wide, with about 70 facets.

Incomplete interocular sutures.

Flagellomeres f1 (= AIII) = 256.28 μm, f2 (= AIV) = 105.03 μm, f3 (= AV) = 111.34 μm, f12 (= AXIV) = 74.36 μm, f13 (= AXV) = 61.80 μm, f14 (= AXVI) = 52.76 μm.

Flagellomere 1 longer than f2+f3. Presence of two ascoids from f1 to f13.

Ascoidal formula: 2/f1- f13 with long ascoids, reaching the next article. One distal papilla on f1, f2 and f3. Absence of papilla from f4 to f11.

One papilla on f11, five on f12, six on f13 and five on f14. No simple setae from f1 to f11.

Three simple setae on f12, four on f13. A total of 18 simple setae observed on f14.

Palps: p1 = 27.75 μm, p2 = 81.06 μm, p3 = 131.03 μm, p4 = 58.39 μm, p5 = 154.01 μm.

Palpal formula: 1, 4, 2, 3, 5

Presence of a group of less than 20 club-like Newstead’s sensilla in the middle third palpal segment of the p3.

No Newstead’s sensilla on other palpal segments.

Presence of one distal spiniform seta on p3, three setae on p4 and eight setae on p5.

Labrum: 187 μm long, f1/E = 1.29.

Hypopharynx with about 15 lateral teeth on either side of the salivary canal. Maxillary lacinia exhibiting eight external and about fifteen internal teeth.

Labial furca closed.

Cibarium armed with four big teeth pointed backwards.

Two groups of about twenty denticles each are observed anteriorly and laterally. Absence of sclerotized area.

Well-developed pharyngeal armature, characteristic of those found on Asian sand fly species of the subgenera *Anaphlebotomus* or *Euphlebotomus*. The armature includes two kinds of teeth: (i) mid-anterior long, dark, and pointing toward the front; (ii) latero-posterior short, in a semi-circular disposition (like an onion bulb).

A discrete pigmentation is observed throughout the pharynx in the center but not laterally nor posteriorly.

**3.3.1.2**. **Cervix**

Two cervical sensilla.

Ventro-cervical sensilla not observable on the holotype.

**3.3.1.3**. **Thorax**

541 μm long.

Light sclerites.

Pleurae: post-alar setae non-observed; presence of two or three proepimeral setae; absence of the upper and lower anepisternal, anepimeral, metaepisternal and metaepimeral setae; a few setae on the anterior region of the katepisternum, and absence of the suture between metaepisternum and katepimeron.

Metafurca with separated vertical arms, mounted in lateral view on all specimens.

Wings: length = 1677 μm, width = 543 μm, r5 = 1063 μm, α (r2) = 402 μm, β (r2 + 3) = 248 μm, δ = 95 μm, γ (r2+3+4) = 189 μm, ε (r3) = 541 μm, θ (r4) = 854 μm, π = 129 μm. Width/γ = 2.87.

Legs:

Anterior leg: coxa = 300 μm; femur, tibia and tarsomeres not observed.

Median leg: coxa = 310 μm; femur, tibia and tarsomeres not observed. Posterior leg: coxa = 343 μm; femur, tibia and tarsomeres not observed.

Abdomen

Tergites ii–vi: presence of setae randomly distributed. Tergite VIII with 25 to 26 setae on each side.

Tergite IX without any protuberance.

Cerci: 152 μm long.

Setae not observed on the X sternite.

**3.3.1.4**. **Genitalia**

Thick wall common duct 508 μm long slightly sclerotized at the base continuing with short narrow thin wall individual ducts 69 μm long.

Spermathecae: 63 μm long and about 15 μm width. They are ringed with more than 15 rings and present a sessile head carried by a broad process.

Genital fork with thick stem over its entire length.

#### 3.3.2. Description of the male of *Ph*. *shadenae n*. *sp*. (Fig 3)

**Fig 3:**
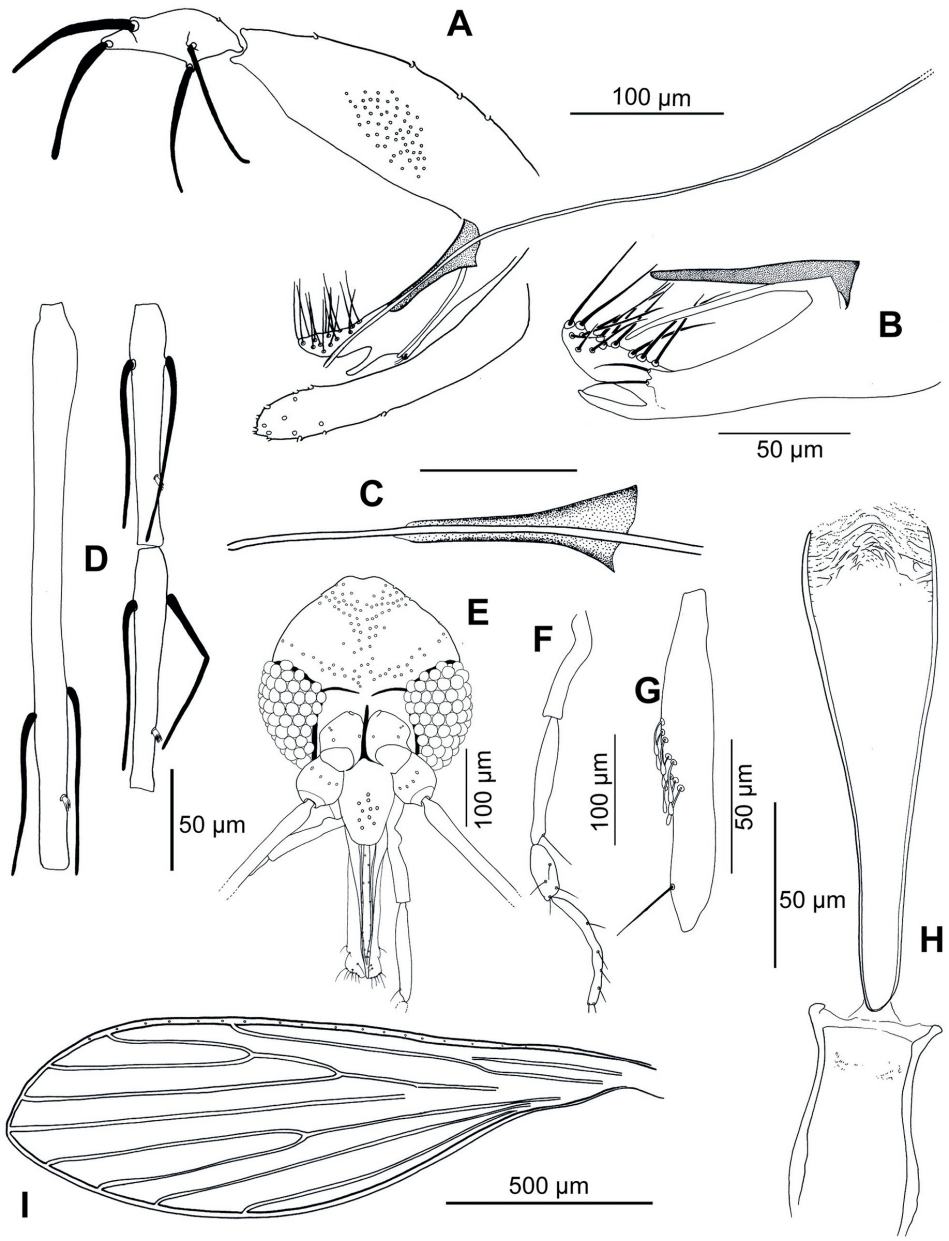
*Phlebotomus shadenae* n.sp. male. A: genitalia (paratype TH19-160); B: detail of the parameral sheath, paramere and intermediate stick (paratype TH19-677); C: detail of the parameral sheath and the distal top of the genital ducts (paratype TH19-160); D: flagellomeres 1, 2 and 3 (= AIII, AIV and AV) (paratype TH19-160); E: head (paratype TH19-160); F: palp (paratype TH19-677); E: third segment of the palp (P3) (paratype TH19-677); H: pharynx and cibarium (paratype TH19-160); I: wing (paratype TH19-677).

The counts and measurements provided below are those of the paratype (allotype) (voucher TH19-160). Comparative measurements of *Ph*. *shadenae* n. sp. with those of *Ph*. *stantoni*, *Ph*. *hoepplii* and *Ph*. *colabaensis* are indicated in Table 4.

**Table 4:** Asian *Anaphlebotomus* male measurements (in μm). Those of *Ph*. *stantoni*, *Ph*. *shadenae* n. sp. and *Ph*. *maynei* were done during the present study. Those of *Ph*. *hoepplii* were calculated from the original description of Tang & Maa [14]. Those of *Ph*. *colabaensis* were extracted from the original description of Mc Combie & Chalam [15].

		*Ph*. *stantoni* (n = 11)				*Ph*. *shadenae n*. *sp*. (n = 3)				*Ph*. *maynei* holotype	*Ph*. *hoepplii* (n = 5)				*Ph*. *colabaensis* holotype
		mean	minimum	maximum	standard deviation	mean	minimum	maximum	standard deviation		mean	minimum	maximum	standard deviation	
Head	length	326.11	283.48	351.44	18.39	315.28	299.7	338.24	20.3						
	width	284.55	259.37	308.38	15.77	264.35	256.2	273.9	8.93						
Clypeus	length	97.32	63.43	115.65	16.79	103.03	93.5	113.48	10.02						110
Flagellomeres	f1	277.44	257.96	302.52	14.96	259.12	242.5	269.55	14.55	274	272.8	262	280	7.53	220
	f2	105.73	96.74	115.87	6.03	111.67	108.3	116.9	4.59		108.6	105	114	4.93	100
	f3	108.26	95.85	121.82	8.41	112.94	106.1	117.83	6.1		108.6	105	114	4.93	100
Labrum	length	168.89	147.72	200.3	20.88	166.88	158.04	181.2	12.51	164					
Palpi	p1	23.21	20.7	26.72	3.13	23.21	20.7	26.72	3.13	28.5	25.6	22	27	2.19	30
	p2	77.92	72.6	85.05	6.42	77.92	72.6	85.05	6.42	68.8	77.4	76	81	2.07	80
	p3	105.65	101.24	109.5	4.16	105.65	101.24	109.5	4.16	125	108.6	97	117	7.50	120
	p4	56	53.9	58.1	2.97	56	53.9	58.1	2.97	58.2	48.4	45	54	3.78	60
	p5	145.14	127.12	163.25	25.46	103.9	88.4	119.4	21.92	145.3	134.6	124	146	10.09	120
Wing	length	1530.60	1408.55	1634.68	68.07	1442.37	1280.62	1533.9	140.49	1543	1677.8	1575	1750	70.97	1470
	width	463.61	399.45	509.92	36.04	483.53	470.2	494.9	12.47	488	495	464	525	25.87	410
Genitalia	gonocoxite	190.03	175.81	206.69	10.20	207.02	205.6	208.4	1.4	217	192.8	171	207	14.74	230
	gonostyle	77.11	71.62	84.04	4.49	84.30	82.5	86.99	2.38	92	84.2	79	90	4.15	150
	parameral sheath	87.98	62.78	110.86	14.94	117.46	101.3	149.49	27.74	100	102.8	90	108	7.95	150
	paramere	139.01	123.95	155.35	11.66	139.76	122.89	171.3	27.33	162	141	117	177	26.5	210
	sperm pump	142.91	126.64	159.29	9.04	139.41	138.26	140.55	1.62	154					
	ejaculatory apodeme	106.93	81.97	124.1	13.53	107.41	105.8	109.02	2.28	121					
	epandrial lobes	180.09	169.71	196.56	7.61	204.14	199.4	207.04	4.14	220					270
	aedeagal ducts	304.20	280.36	335.65	18.54	655.47	639.9	671.04	22.02	448					
	sperm pump + aedeagal ducts	419.46	146.34	478.44	93.45	703.17	688	718.34	21.45	602	411.2	376	420	19.68	
	internal gonocoxal tuft setae	33.09	23	43	7.18	48.33	44	56	6.66	49					

**3.3.2.1**. **Head**

Occiput with two lines of well individualized setae.

Clypeus: 113 μm long and 59 μm wide with nine big setae randomly distributed.

Eyes: 153 μm high, 83 μm wide, with about 80 facets.

Interocular sutures incomplete. Interantennal sutures do not reach the interocular ones.

Flagellomeres: f1 (= AIII) = 269.55 μm, f2 (= AIV) = 116.90 μm, f3 (= AV) = 117.83 μm, f12 (= AXIV) = 43.96 μm, f13 (= AXV) = 47.11 μm, f14 (= AXVI) not observed.

Ascoidal formula: 2/f1-f13. Presence of two long ascoids, reaching the next articulation on f1 and 2 long but shorter ascoids on f2 and f3.

One distal papilla on f1, f2 and f3. Two papillae on f11, seven on f12, and six on f13 and f14.

No simple setae from f1 to f8. One simple seta on f8 and f9. Two simple setae on f10 and f11. Three simple setae on f12, two on f13 and about 10 on f14.

Palpi: p1 = 26.72 μm, p2 = 85.05 μm, p3 = 101.24 μm, p4 and p5 not observed on the allotype TH19- 160.

Palpal formula: 1, 4, 2, 3, 5 calculated on allotype TH19-677.

Presence of a group of about 10 club-like Newstead’s sensilla in the middle of the third palpal segment.

Lack of Newstead’s sensilla on other segments.

Presence of one distal spiniform seta on p3, four on p4 and less than 10 on p5 (on allotype TH19- 677)

Labrum: 158 μm long, f1/ labrum = 1.71.

Labial furca closed.

Cibarium: presence of a few small lateral teeth (denticles), mostly grouped in packs of three to four.

Absence of a sclerotized area.

Pharyngeal armature takes up the entire fourth or fifth posterior part of the pharynx with small teeth arranged concentrically. The central and anterior teeth are concentric translucent lines whereas the posterior and lateral teeth are dot-like and grouped in concentric rows.

**3.3.2.2**. **Cervix**

Two cervical sensilla on each side. One ventro-cervical sensillum.

**3.3.2.3**. **Thorax**

466 μm long.

Poorly pigmented sclerites. Mesonotum: absence of a post-alar seta.

Pleurae: three proepimeral setae on each side: two basal and one upper; post-alar setae non-observed; absence of the upper and lower anepisternal, anepimeral, metaepisternal and metaepimeral setae; absence of setae on the anterior region of the katepisternum, and absence of the suture between metaepisternum and katepimeron.

Metafurca with separated vertical arms.

Wings: Length = 1280 μm, width = 485 μm, r5 = 1020 μm, α (r2) = 347 μm, β (r2 + 3) = 241 μm, δ = 40 μm, γ (r2+3+4) = 184 μm, ε (r3) = 501 μm, θ (r4) = 797 μm, π = 136 μm. Width/γ = 2.64.

Legs:

Anterior leg: coxa = 305 μm; femur, tibia and tarsomeres not observed.

Median leg: coxa = 310 μm; femur, tibia and tarsomeres not observed.

Posterior leg: coxa = 320 μm; femur, tibia and tarsomeres not observed.

**3.3.2.4**. **Abdomen**

Tergite ii: presence of randomly distributed setae.

The others tergites are not observable since they were used for the molecular analyses.

**3.3.2.5**. **Genitalia**

Absence of abdominal rods.

Gonocoxite with a median tuft of 56 internal setae. Absence of basal gonocoxal lobe.

Gonostyle with four thick spines: one terminal, one subterminal and two basal ones. Absence of accessory setae.

Complex paramere, with a long upper lobe with about 20 strong upward-facing setae, an intermediate shorter and narrow hairless lobe, and a very small lower lobe exhibiting two setae.

Presence of a long, narrow and translucent accessory spine between the paramere and the parameral sheath.

Parameral sheath finger-like.

Aedeagal ducts straight, slender throughout, tapering evenly throughout with a round end. Very long smooth aedeagal filaments, isodiametric.

Epandrial lobes about as long as the gonocoxites, without permanent setae.

Cerci: 127 μm long.

#### 3.3.3. Description of the female of *Sergentomyia maiae* Depaquit, Renaux Torres and Siriyasatien n. sp. (Fig 4)

**Fig 4:**
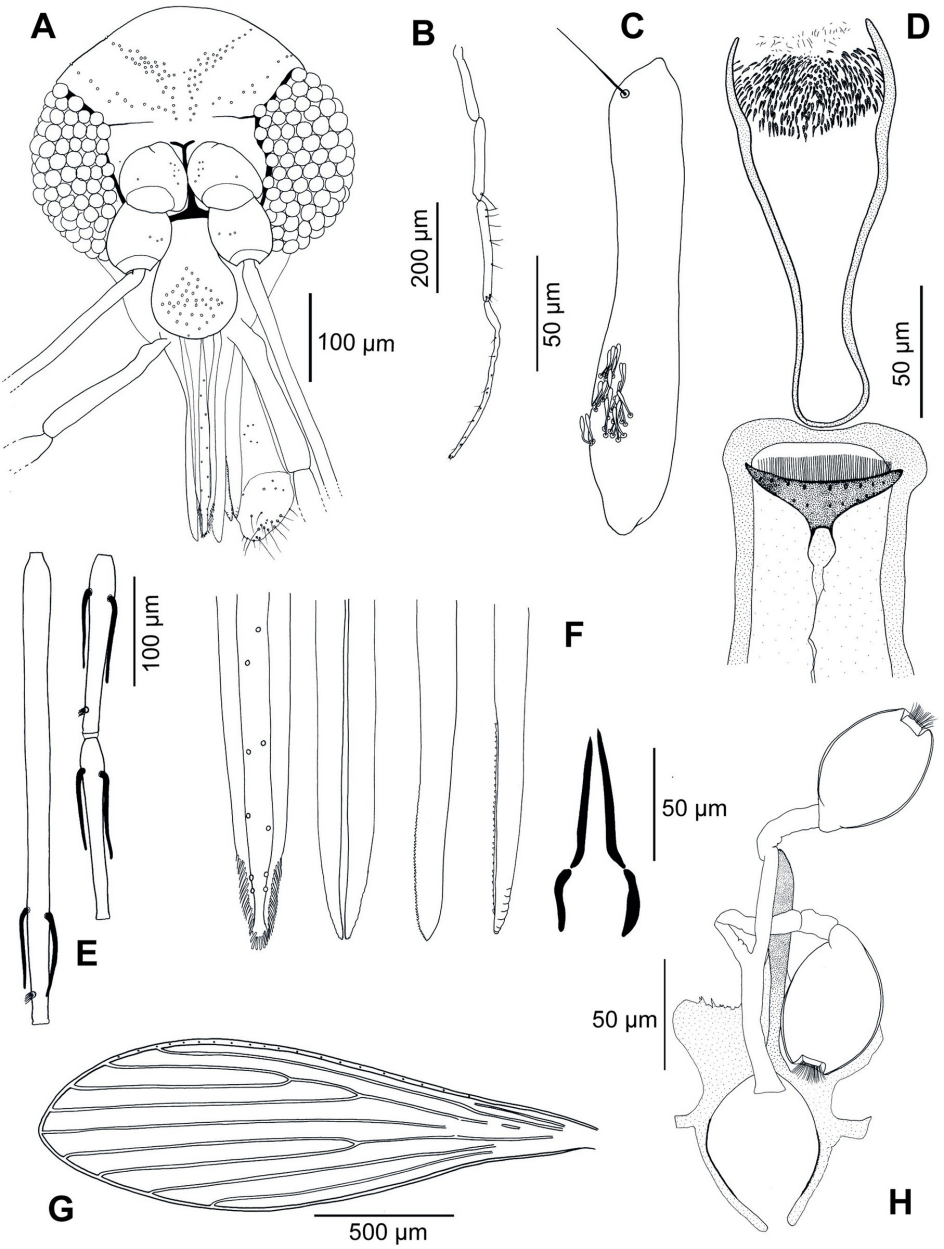
*Sergentomyia maiae* n.sp. Female. A: head (holotype); B: palp (holotype); C: third segment of the palp (P3) (paratype TH19-572); D: pharynx and cibarium (paratype TH19-148); E: flagellomeres 1, 2 and 3 (= AIII, AIV and AV) (paratype TH19-572); F: mouth parts (labrum, hypopharynx, mandible, maxilla, and labial furca from left to right) (paratype TH19-148); G: wing (holotype); H: furca and spermathecae (paratype TH19-572).

Genus: *Sergentomyia*

Subgenus: ungrouped, awaiting a taxonomic revision of the genus *Sergentomyia*.

Measurements indicated in the main text are those of the holotype (voucher TH19-756). Measurements carried out on other specimens are indicated in Table 5.

**Table 5:** *Sergentomyia maiae* n. sp. female measurements (in μm). n = 12.

		mean	minimum	maximum	standard deviation
Head	length	376.05	318.7	407.44	27.26
	width	337.36	252.28	378.71	39.34
Clypeus	length	150.30	100.45	166.73	19
	width	76.87	60.79	104.66	15.7
Eyes	length	198.54	183.36	217.89	12.6
	width	105.27	81.25	120.27	12.91
Flagellomeres	f1	372.08	330.08	418.38	27.8
	f2	142.95	129.11	161.1	9.61
	f3	148.91	132.57	163.35	9.74
Labrum	length	213.115	184.37	244.52	18.21
Palpi	p1	36.43	30.98	43.68	4.63
	p2	125.51	107.14	139.25	10.43
	p3	178.88	150.49	202.47	15.76
	p4	209.26	177.45	252.58	21.9
	p5	362.57	306.0	413.37	36.14
Cibarium	teeth	62.5	57.0	69.0	5.5
	vertical teeth	9.4	8.0	13.0	2.07
Wing	length	1910.30	1549.62	2203.24	204.41
	width	581.86	349.69	725.87	107.3
	r5	1346.3	814.19	1547.09	205.44
	r2	618.48	217.45	745.7	170.91
	delta	445.48	88.37	559.54	152.52
	r2+3	248.31	213.02	270.21	17.93
	r3	740.42	338.48	886.07	164.26
	r2+3+4	289.05	163.05	347.95	49.64
	r4	1051.56	634.68	1214.51	173.51
	pi	39.3	30.04	49.41	7.33
Spermathecae	length	46.06	39.34	56.44	7.61
	width	19.18	13.69	31.5	7.28

***Etymology***: The epithet *maiae* refers to our colleague Carla Maia who was with us in Thailand when we collected these sand flies.

**Type locality**: Pha Thong cave (20° 5′28.89"N, 99°54′13.09"E), Chiang Rai province, northern Thailand.

**Type specimens**. Holotype female (voucher TH19-756) and two female paratypes (vouchers TH19-139 and TH19-148) deposited at the Laboratory of Entomology of the Muséum National d’Histoire Naturelle de Paris, France under identification numbers MNHN-ED-ED11204, MNHN-ED-ED11205, and MNHN-ED-ED11206.

Two female paratypes (vouchers TH19-572 and TH19-621) deposited at the Natural History Museum of the National Science Museum, Thailand, under identification numbers THNHM-I-00028013 and THNHM-I-00028014.

**Note**: The authors of the new taxa are different from the authors of this paper: Article 50.1 and Recommendation 50A of the International Code of Zoological Nomenclature [13].

**3.3.3.1**. **Head**

Occiput with two narrow lines of well individualized setae.

Clypeus: 163 μm long and 61 μm wide with 39 setae randomly distributed. Eyes: 201 μm long, 120 μm wide, with about 90 facets.

Interocular sutures incomplete.

Interantennal sutures do not reach the interocular sutures.

Flagellomere: 1 longer than f2+f3.

Presence of two ascoids from f1 to f11 and one or two on f12 and f13 and one on f14.

Ascoidal formula: 2/f3- f13 with long ascoids, not reaching the next article.

One papilla on f1 and f2. Absence of papilla from f3 to f7.

One median papilla on f7 and f8, two on f9 (one anterior and one median), six on f10 and f11, seven on f12, six on f13 and four on f14.

No simple seta on f1, one on f2 and f3, two on f4 and f5, three on f6 to f8, two on f7, three on f8, five on f9, four on f10, four on f11, four on f12 (one median and three distal), three on f13 and less than ten on f14.

Palpal formula: 1, 2, 3, 4, 5.

Presence of a group of about 30 club-like Newstead’s sensilla implanted proximally on the third palpal segment.

Presence of one distal spiniform seta on p3 and six on p4. Labrum: 202 μm long.

Hypopharynx with about 10 faint teeth on either side of the salivary canal. Maxillary lacinia exhibiting eight external teeth and about thirty internal teeth.

Labial furca opened.

Cibarium armed with 59 parallel and palisade-like light teeth organized along a straight and horizontal line.

One row of nine big denticles on a line just below the teeth. One row of less than 10 teeth implanted more basally, in the very wide and pigmented sclerotized area. Russula mushroom-like.

Pharynx strongly armed on its posterior quarter with monomorphic pigmented teeth each being *Se*. *antennata*-like. A discrete constriction is observed at the level of the anterior pharyngeal teeth.

**3.3.3.2**. **Cervix**

Two cervical sensilla on each side.

Two ventro-cervical sensilla were observed on paratype TH19-139.

**3.3.3.3**. **Thorax**

Light brown sclerites.

Mesonotum: absence of post-alar seta.

Pleurae: Proepimeral setae non-observed; absence of the upper and lower anepisternal, anepimeral, metaepisternal and metaepimeral setae; absence of seta on the anterior region of the katepisternum, and absence of the suture between metaepisternum and katepimeron.

Metafurca with separated vertical arms.

Wings:

Length = 1952 μm; Width = 597 μm.

r5 = 1419 μm, α (r2) = 647 μm, β (r2 + 3) = 260 μm, δ = 476 μm, γ (r2+3+4) = 314 μm, ε (r3) = 786 μm, θ (r4) = 1087 μm, π = 30 μm. Width/γ = 1,90.

Legs (on TH19-148):

Anterior leg: coxa = 358 μm; femur, tibia and tarsomeres not observed.

Median leg: coxa = 339 μm; femur, tibia and tarsomeres not observed.

Posterior leg: coxa = 388 μm; femur, tibia and tarsomeres not observed.

**3.3.3.4**. **Abdomen**

Setae randomly distributed on the first tergite.

**3.3.3.5**. **Genitalia**

Smooth elongated capsule-like spermathecae. Thick walls. Capsule 31 μm wide and 60 μm long. Short terminal knob embedded in the capsule.

Smooth ducts.

Genital furca with two well developed lateral processes.

## Discussion

The species *Phlebotomus shadenae* n. sp. belongs to the subgenus *Anaphlebotomus* because the male exhibits four spines on the gonostyle and the gonocoxite lacks a basal lobe. The female exhibits a pharyngeal armature including two kinds of teeth and cibarial teeth which are typical of this subgenus (also shared by some species of the subgenera *Euphlebotomus* and *Madaphlebotomus*).

*Ph*. *shadenae* is very close to *Ph*. *stantoni* Newstead, 1914, of which it differs for both gender due to the length of the aedeagal ducts and by the number of gonocoxal setae for the male, and by the length of the spermathecal ducts for the female.

We only have molecular data for a single female but it clearly supports its individualization compared to the many *Ph*. *stantoni* that were sequenced in our study (Fig 1 and Table 2).

The attachment of male to female is based on the resemblance in both gender to *Ph*. *stantoni* and by the sharing of long aedeagal ducts which are compatible with the long length of the spermathecal ducts.

*Phlebotomus stantoni* is by original designation, the type-species of the subgenus *Anaphlebotomus*. The genus *Phlebotomus* has long been described as possessing a group of setae on the lower anepisternum. In light of the standard for the description of new species [12], an in-depth examination of this thoracic region shows the absence of anepisternal setae and the presence of two to three proepimeral setae, unlike Abonnenc who described them in *Ph*. *stantoni* [16].

Before the present study, this subgenus included five species according to Seccombe et al. [17]: *Ph*. *stantoni*, *Ph*. *colabaensis* Mc Combie, Young & Chalam, 1927, *Ph*. *rodhaini* Parrot, 1930, *Ph*. *rousettus* Davidson, 1981 and *Ph*. *fortunatarum* Morillas-Marquez, Castillo-Remiro & Ubeda-Ontiveros, 1984. Over time, the subgenus *Abonnencius* Morillas-Marquez, Castillo-Remiro & Ubeda-Ontiveros, 1984, previously wrongly synonymized with *Anaphlebotomus* by Lane & Alexander [18], was reinstated [19, 20]. Consequently, *Ph*. *fortunatarum* was excluded from the subgenus *Anaphlebotomus*. In Madagascar, several *Phlebotomus* species include males which have gonostyles exhibiting four spines and a gonocoxite without basal process. Based on these characteristics, these sand flies species were provisionally classified in the subgenus *Anaphlebotomus* [21–25] pending the description of the subgenus *Madaphlebotomus* Depaquit, Léger & Randrianambinintsoa, 2013, including all the Malagasy *Phlebotomus* described in the genus *Anaphlebotomus* up to 2013 [26]. In this paper, we do not include three Indian species in the subgenus *Anaphlebotomus*, respectively: *Ph*. *sanctijohani* Ipe & Singh 1994, *Ph*. *palamauensis* Singh, Phillips-Singh & Ipe 2007, and *Ph*. *chiyankiensis* Singh, Phillips-Singh & Ipe 2009 [27–29]. Without access to the type-specimens, by looking at the descriptions and drawings provided by the authors, the single paramere of the males, the terminal and subterminal position of the spines as well as the presence of an accessory seta on the gonostyle, suggest that these species could all, in fact, belong to the genus *Sergentomyia*. As we wait for future taxonomic studies which will confirm or invalidate the systematic position of these three species, we prefer not to consider them in the present paper. In the future, if their affiliation to the subgenus *Anaphlebotomus* is proved, their individualization would be very easy thanks to the characters mentioned above. In Asia, the distribution of *Ph*. *colabaensis* is limited to India and Pakistan [15, 30] whereas the distribution of *Ph*. *stantoni* is wider. *Ph*. *stantoni* is found from India to China and as far as South-East Asia [17] when taking into account the synonymizing of both the Indian species *Ph*. *maynei* Sinton, 1930, and the Chinese species *Ph*. *hoepplii* Tang & Maa, 1945.

*Phlebotomus stantoni* seems easy to identify because it has very specific characteristics. Paradoxically, this great ease of identification can mask discrete morphological differences which could be very specific. Taking this idea into consideration, in light of the description of a new species close to *Ph*. *stantoni*, it would seem judicious to carry out a careful examination of the literature related to the Asian subgenus *Anaphlebotomus* and to examine the available type-specimens of the species in this group, including those whose synonymy has been proposed in the past.

The validity of *Ph*. *shadenae* n. sp. is based on morphological and molecular arguments. From a morphological point of view, *Ph*. *shadenae* n. sp. differs from *Anaphlebotomus* by the length of the genital filaments and by the length of the spermathecal ducts as well as by the number of setae borne on the gonocoxite. Furthermore, the molecular data individualize *Ph*. *stantoni* and *Ph*. *shadenae* n. sp. robustly.

The morphological analysis of a collection of sand flies caught in Laos during the present study revealed that other specimens of *Ph*. *shadenae* n. sp. were recorded in Laos. Moreover, according to nucleotide sequences, *Ph*. *shadenae* n. sp. seems also to have been recorded in Southern Thailand a few years ago, though they were not identified correctly: a nucleotide blast indicated that the sequence of the *Ph*. *shadenae* n. sp. specimen processed in the present study is identical to that of the specimen labelled *Ph*. *stantoni* deposited by Hirotomo, Tiwananthagorn and Siripattanapipong under the Genbank accession number MG770905. This specimen seems to have been excluded from the study published by Siripattanapipong et al. [31] including only Genbank records ranging from MG770906 to MG770935. Consequently, the distribution area of *Ph*. *shadenae* includes the north and the south of Thailand as well as Laos. A careful identification of the species of the subgenus *Anaphlebotomus* is required in the future throughout South-East Asia.

The validity of *Ph*. *colabaensis* Young & Chalam, 1927 is accepted by all the authors and is based on the morphology of the spermathecae (Sinton, 1932) or on several parts of the male genitalia (gonostyle, parameral sheath, paramere, epandrial lobes) as indicated in Table 4.

The synonymy of *Ph*. *hoepplii* Tang & Maa, 1945 is difficult to validate without accessing the type-specimens. It has been considered as a junior synonym of *Ph*. *stantoni* by Lewis [32] based on the examination of “drawings made by Raynal and by Raynal and Gaschen” without examining the type-specimen, whereas Leng considered it as a valid species [33]. It is unclear whether the specimens drawn were really *Ph*. *hoepplii* or not. However, following a careful examination of the description of *Ph*. *hoepplii* [14], we consider this synonymy as erroneous and we consider *Ph*. *hoepplii* as a valid species. In our opinion, the very short individual spermathecal ducts of this species have never been observed in *Ph*. *stantoni* and this character supports in and by itself the validity of this species.

**Fig 5:**
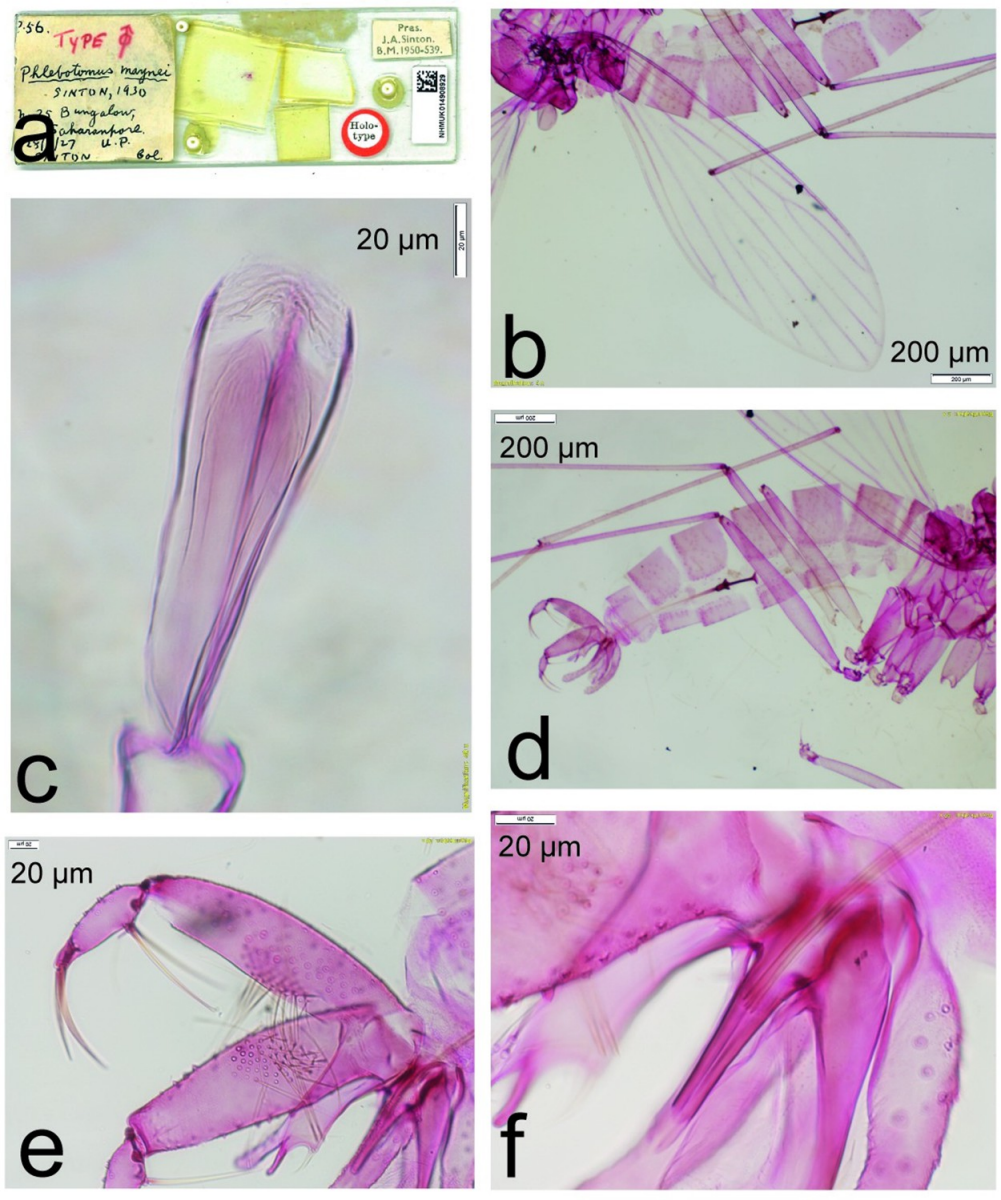
*Phlebotomus maynei* male holotype NHMUK014908929. a: slide; b: wing; c: pharynx and cibarium; d: abdomen and genitalia; e: detail of the genitalia; f: parameral sheath with genital ducts. Courtesy of the Natural History Museum, London.

Another closely related species from Saharanpur, Uttar Pradesh, North of India, was described: *Ph*. *maynei* Sinton, 1930 [34]. This species has been considered as a junior synonym of *Ph*. *stantoni* by Raynal & Gaschen [35]. In this previous study, which described for the first time the male of *Ph*. *stantoni*, the authors noticed the important similarities of the genitalia of *Ph*. *stantoni* and *Ph*. *maynei*. Indeed, at the time, a few species of sand flies were described and more subtle variations such as the length of the genital filaments did not appear to be ultimately different. In addition to the allopatric distribution of these two species, it seems obvious to us that the length of the genital filaments of *Ph*. *maynei*, which is of intermediate length between that of *Ph*. *stantoni* (short) and that of *Ph*. *shadenae* n. sp. (long), is a relevant specific character. It supports our idea that *Ph*. *maynei* needs to be considered as a valid species henceforth (Fig 5). In fact, we consider without any overlap in the distribution of a character like the length of aedeagal and spermathecal ducts as specific because they have to be more of less similar for an efficient mating in natural conditions.

A careful attention must be paid to the potential vector role of *Ph*. *stantoni* and possibly its closely related species. Recently, DNA of an unknown *Trypanosoma* sp. was detected from a *Ph*. *stantoni* in Thailand [36]. In addition, Preativatanyou et al. *Trypanosoma* sp. DNA as well as *Leishmania donovani* complex DNA in *Ph*. *stantoni* from Southern Thailand [37].

Regarding the other *Phlebotomus* found in the present study, we have caught a few specimens of *Ph*. *barguesae* and *Ph*. *mascomai*, both cavernicolous species described from Thai caves [38, 39]. The first species has been recorded from another cave in Thailand [2] whereas the latter has been found recently in some caves in Thailand [3, 40] as well as in Vietnam [41] showing these species seems widely distributed in South-Eastern Asian caves.

*Idiophlebotomus longiforceps* has been described from caves in the South of China under the genus *Sergentomyia* [42]. It has also been recorded in caves in Thailand and Vietnam [3, 43]. To our knowledge, no record of this species has been done outside caves, as well as for the genus *Chinius*. In the present study, we have found *Ch*. *barbazani*, a species only recorded in Thai caves [2, 44, 45]

*Sergentomyia maiae* n. sp. is described from the female. The male remains unknown though we processed several *Sergentomyia* spp. males for cytochrome b sequencing, but their sequences did not match with those of female *Se*. *maiae*. Regarding the forked sclerotized area and the cibarial and pharyngeal armatures, this species is close to the *Se*. *barraudi* group. The taxonomy of this group is unresolved as of yet. As indicated, the *Se*. *barraudi* group should be further studied because of considerable heterogeneity in the morphological characters, such as the number and distribution of teeth on the cibarium [41]. In the original description made on Indian specimens, Sinton counted 40 cibarial teeth. Regarding the molecular data, there is also an important variability within *Se*. *barraudi* (Fig 1). *Sergentomyia maiae* n. sp. exhibits 57 to 69 cibarial teeth. It is different from other members of *Se*. *barraudi* because there is a completely original double row of vertical teeth, and particularly because of the very low position of one of these rows of teeth. The row closest to the cibarial teeth include eight to 13 vertical teeth whereas the other row includes six to nine vertical teeth.

**Fig 6:**
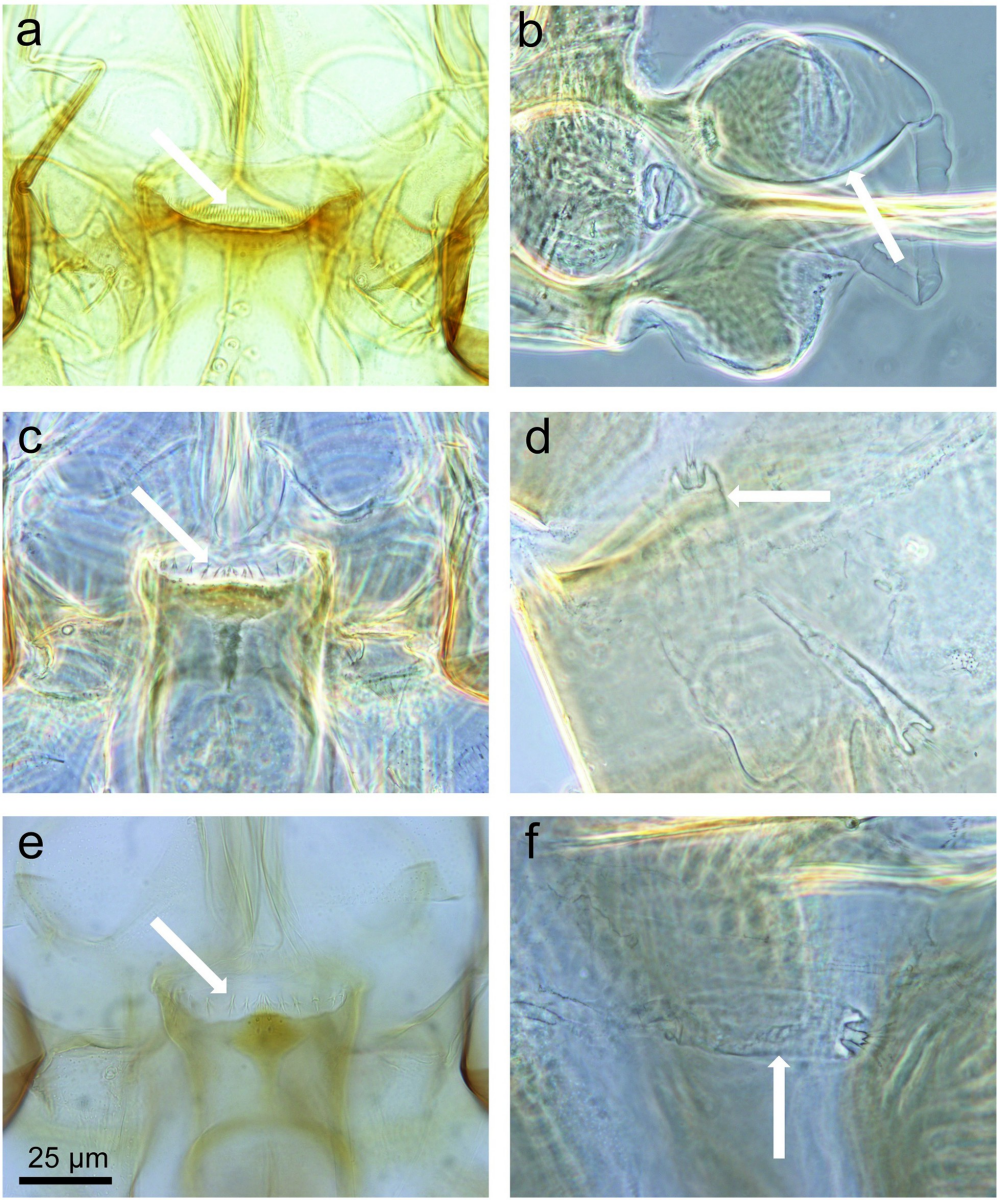
Cibariums (left column) and spermathecae (right column) of *Sergentomyia spp*. *Sergentomyia siamensis* (a, b), *Sergentomyia khawi* (c, d), and *Sergentomyia cf iyengari* Raynal-like specimen TH19-507 (e, f). White arrows show the cibarial teeth (on the left) and the spermathecae (on the right).

The other members of the *Se*. *barraudi* group processed in the present study (Fig 6) exhibit 44 to 54 cibarial teeth (mean 47.3, standard-deviation 3.37) meaning these are quite different from *Se*. *barraudi* s. st. In view of the different biogeographical subzones in the Indo-Malaysian area, it is logical to assume that the populations from Thailand could be individualized from those from India, and possibly belong to different species. In our opinion, the specimens we processed belong to the species *Se*. *siamensis* Causey precisely described from Thailand and exhibiting 44 cibarial teeth according to the original description [46].

Regarding the *Se*. *iyengari* group, most of the specimens we processed from Pha Tong cave do not belong to *Se*. *iyengari* s. st. described from the South of India, without vertical teeth under the cibarial teeth. We think our specimens could belong to *Se*. *khawi*, as they exhibit many vertical teeth arranged along 2–3 rows, though they have less teeth (13–17) than in the original description by Raynal [47] from Chinese specimens. Moreover, as shown in Table 1, we also found a female which is morphologically identical to the one described by Raynal & Gaschen as *Se*. *iyengari* [48]. However, we think that this species is not related to *Se*. *iyengari* but to another probable new species which has yet to be described. In this study, we named this female sand fly *Se*. *cf iyengari* Raynal-like, pending further studies (Fig 6, Table 1). There is a great need to revise the *Se*. *iyengari* group by comparing several populations from India, China and South-Eastern Asia using morphological and molecular approaches.

*Sergentomyia hivernus* is one of the species historically wrongly considered junior synonyms of *Se*. *iyengari*. It is now considered as a valid species which can be identified thanks to its wide spermatheca and to the low number of vertical teeth of the cibarium [36].

*Sergentomyia sylvatica* was first described from Vietnam outside [49] and the specimens collected in the present study are morphologically similar to those originally described: a few teeth in the center of the cibarium and spermathecae not completely segmented.

As indicated by Vu et al. working on sand flies from Vietnam [41], *Se*. *anodontis* could include several populations taking into account the exhibition of any lateral teeth or not. These populations are everytime caught in caves [3, 4, 6, 40, 45, 50–52]

The identification of the *Grassomyia* of the present study seems not possible at a specific level. A complete revision of this genus has to be carried out. Known to live in open biotopes i.e. savannah [53], it is surprising to catch them in a cave.
